# Wnt/TLR Dialog in Neuroinflammation, Relevance in Alzheimer’s Disease

**DOI:** 10.3389/fimmu.2017.00187

**Published:** 2017-02-24

**Authors:** Juan M. Zolezzi, Nibaldo C. Inestrosa

**Affiliations:** ^1^Centro de Envejecimiento y Regeneración (CARE-UC), Facultad de Ciencias Biológicas, Departamento de Biología Celular y Molecular, P. Universidad Católica de Chile, Santiago, Chile; ^2^Centre for Healthy Brain Ageing, Faculty of Medicine, School of Psychiatry, University of New South Wales, Sydney, NSW, Australia; ^3^Centro de Excelencia en Biomedicina de Magallanes (CEBIMA), Universidad de Magallanes, Punta Arenas, Chile

**Keywords:** immune response, neuroinflammation, Alzheimer’s disease, toll-like receptors, Wnt signaling

## Abstract

The innate immune system (IIS) represents the first line of defense against exogenous and endogenous harmful stimuli. Different types of pathogens and diverse molecules can activate the IIS *via* a ligand–receptor mechanism. Cytokine release, recruitment of immunocompetent cells, and inflammation constitute the initial steps in an IIS-mediated response. While balanced IIS activity can resolve a harmful event, an altered response, such as deficient or persistent IIS activity, will have a critical effect on organism homeostasis. In this regard, chronic IIS activation has been associated with a wide range of diseases, including chronic inflammatory disorders (inflammatory bowel disease, arthritis, chronic obstructive pulmonary disease, among others), cancer and, more recently, neurodegenerative disorders. The relevance of the immune response, particularly inflammation, in the context of neurodegeneration has motivated rigorous research focused on unveiling the mechanisms underlying this response. Knowledge regarding the molecular hallmarks of the innate immune response and understanding signaling pathway cross talk are critical for developing new therapeutic strategies aimed at modulating the neuroinflammatory response within the brain. In the present review, we discuss the IIS in the central nervous system, particularly the cross talk between the toll-like receptor-signaling cascade and the wingless-related MMTV integration site (Wnt) signaling pathway and its relevance in neurodegenerative disorders such as Alzheimer’s disease.

## Central Nervous System (CNS), Alzheimer’s Disease (AD), and the Immune Response: General Considerations

The CNS constitutes a highly specialized structure with neuronal physiology depending on the fulfillment of specific microenvironmental requirements, which are necessary to control the molecular and cellular efflux/influx between the CNS and blood (1–3). This controlled isolation, which is mainly established by the blood–brain barrier (BBB), initially led to the idea that the CNS was deprived of immune cells, although its ability to respond to systemic and localized pathological conditions have not only demonstrated its capacity to exhibit an immune response but has also allowed the identification of specific cells that are responsible for this function (4). Within the brain, microglia and astrocytes are specialized cells that function in immune surveillance and response against several pathological conditions (4–8). Importantly, common systemic immune cells, such as cluster of differentiation 11b and c (CD11b, CD11c)-positive cells, can be found in the CNS during the brain immune response, and these factors mainly localize close to BBB-damaged areas, suggesting that their migration from the periphery and colonization of the CNS are due to an increase in BBB permeability (4, 9–12). However, it has been recognized that the brain parenchyma constitutes an anti-inflammatory environment that is rich in anti-inflammatory mediators, such as transforming growth factor β (TGFβ) and interleukin (IL)-10, which prevent peripheral immune cell proliferation (13–15), providing further support to the hypothesis that glial cells within the brain are responsible for the IIS response. Although it is possible that these characteristics are evidence of an immune-incompetent organ, Ransohoff and Brown (15) proposed that these particularities appear to be necessary to avoid strong immune responses, which can further damage neurons. Consequently, even when the protective role of the IIS is not considered, the balance of the immune response is critical within the CNS to prevent additional damage. Importantly, this balance is dependent on both signals to unleash the immune response. However, how CNS competent cells interpret these signals, including the molecular mechanisms triggered as a part of this response, remains to be determined.

The immune response and the inflammatory component of such a response are most relevant in several neurodegenerative disorders, including AD, Parkinson’s disease, Huntington’s disease, multiple sclerosis, and amyotrophic lateral sclerosis. Indeed, the broad molecular alterations verified during the course of these pathologies have identified the inflammatory response as a critical element for consideration at different stages of the disease. Consistent with these findings, many efforts have committed not only to understand the inflammatory response within the brain or the CNS but also to identify new molecular targets that can modulate the inflammatory process. Thus, in the present review, we describe some of the particularities of the toll-like receptors (TLRs) and the molecular mechanisms triggered after their activation, as well as their close relationship with the wingless-related MMTV integration site (Wnt) signaling pathway. Similarly, we summarize some of the relevant scientific evidence suggesting that the cross talk between TLR and Wnt signaling plays a relevant role in neurodegenerative disorders, particularly in AD.

### AD and Neuroinflammation

According to Alzheimer’s Disease International, AD is the leading cause of dementia, accounting for up to 50–75% of all dementia cases worldwide. With a total estimated cost of up to $818 billion, AD is a worldwide public health issue (https://www.alz.co.uk/research/WorldAlzheimerReport2015.pdf). Many human and financial efforts have been committed to understanding this pathology in an attempt to prevent, slow, stop, and/or reverse AD, which was first described in the early 1900s. Although relevant progress has been made through the years, our knowledge remains incomplete, and no effective therapy is currently available to treat this pathology. Clinically, AD is characterized by progressive cognitive impairment with mood and behavioral alterations. Memory loss worsens as pathology progresses, reflecting brain atrophy as a consequence of severe neuronal loss (16–20). However, extracellular amyloid-β peptide (Aβ) aggregates, also known as senile plaques, and intracellular neurofibrillary tangles (NFTs) consisting of hyperphosphorylated *tau* protein are the molecular hallmarks of the disease and are considered the basis of the molecular alterations leading to neuronal death (20–22). Moreover, according to the amyloid hypothesis of AD, Aβ constitutes the starting point for all alterations observed during disease progression, including the NFTs, and although the triggering mechanisms leading to increased Aβ production and aggregation have not yet been elucidated, our understanding of the Aβ effects on the cell molecular machinery has improved significantly (23–27). Increased production of reactive oxygen species (ROS), mitochondrial dysfunction, NFT formation, increased Aβ production, and synaptic disruption are some of the consequences of exposure to Aβ (28, 29). However, in recent years, the Aβ-related inflammatory component of the pathology has become significantly relevant and is considered a critical target to control AD (30). Moreover, it has been suggested that permanent exposure to Aβ due to an increased production or a deficient clearance mechanism from the brain will lead to a chronic inflammatory state, which results in a harmful environment for the neurons, causing additional damage and ultimately further neuronal death (3, 30). Furthermore, the inflammatory mechanisms triggered by Aβ are driven mostly through the TLR family.

## Toll-Like Receptors

As the first unspecific defense, the IIS functions to sense both molecular patterns related to pathogenic presence (pathogen-associated molecular patterns, PAMPs) and molecular patterns related to endogenous molecules indicative of cell damage (damage-associated molecular patterns, DAMPs) *via* specific receptors known as pattern recognition receptors (PRRs) (5, 6, 31, 32). The TLR family constitutes a highly relevant type of PRR that is necessary not only to unleash the initial immune response but also to connect this first unspecific defense with secondary adaptive immunity (6). The presence of TLRs has been determined in several cell components of the peripheral immune system and in immunocompetent cells of the brain, such as astrocytes and microglia, as well as in neurons and oligodendrocytes (5, 6), suggesting that each cell type within the brain can sense and react to harmful molecular patterns. It has also been demonstrated, with some discrepancies between studies, that microglia and neurons express all TLR subtypes, while astrocytes express a more limited repertoire, including TLR2, TLR3, TLR4, TLR9, and TLR11 (33, 34).

**Figure 1:**
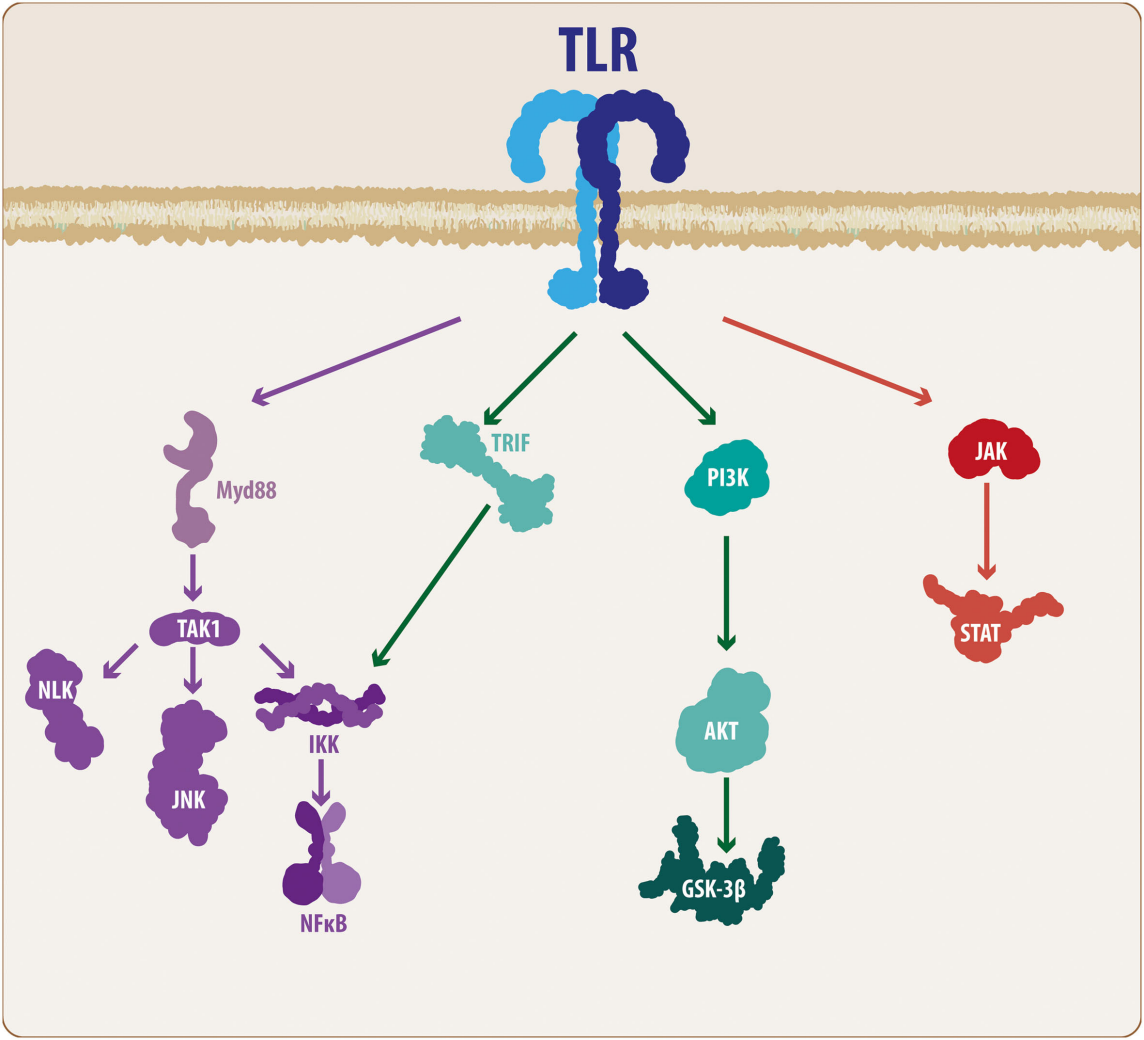
**TLR molecular cascade**. Upon activation, TLRs can signal through different transduction molecules. The MyD88-mediated pathway constitutes the classical TLR signaling pathway and ultimately leads to the activation of the NF-κB transcription factor, resulting in the production and release of pro-inflammatory mediators. In addition, some members of the TLR family can activate other pathways, such as PI3K and JAK/STAT. Although these mechanisms also lead to NF-κB activation, the intermediary molecular nodes can interact and activate additional signaling pathways. TLR, toll-like receptor; MyD88, myeloid differentiation factor 88; TAK1, transforming growth factor-β-activated kinase-1; IKK, inhibitory NF-κB kinases; NLK, nemo-like kinase; JNK, c-Jun N-terminal kinases; NF-κB, nuclear factor-κB; TRIF, TIR-containing adaptor inducing interferon-β; PI3K, phosphatidylinositide-3 kinase; Akt, protein kinase B; GSK3β, glycogen synthase kinase 3 β; JAK, Janus kinase; STAT, signal transducer and activator of transcription.

Several members of the TLR family have been described, ranging from 11 to 13 subtypes and depending on the species (5). According to Hanke and Kielian (6), TLRs can be divided into two main groups: those expressed on the plasma membrane, such as TLRs 1, 2, 4, 5, and 6, and those expressed on endosomes, such as TLR 3, 7, 8, and 9. In addition, TLRs 1, 2, 4, and 6 recruit an adaptor protein known as cluster of differentiation 14 (5, 6). While external TLRs sense bacterial proteins, such as lipopolysaccharide, flagellin and lipoproteins, internal TLRs sense viral components, such as viral RNA and DNA, as well as non-methylated CpG-enriched DNA (6, 15, 31). Importantly, TLRs consist of a toll/interleukin-1 (TIR) intracellular domain, which, once ligand-activated, triggers the molecular cascade necessary for an immune/inflammatory response (5). TLRs usually signal through the myeloid differentiation factor 88 (MyD88) pathway. Accordingly, MyD88 recruitment leads to the activation of interleukin-1 receptor-associated kinase (IRAK) family of proteins, which in turn results in the activation of tumor necrosis factor receptor-associated factor 6, causing the recruitment of transforming growth factor-β-activated kinase-1 (TAK1). TAK1 along with TAK1-binding proteins activate the IKK complex, resulting in the phosphorylation of IκB factor, which induces the release of nuclear factor-κB (NF-κB) and enables its translocation to the nucleus and subsequent expression of inflammatory-related genes (Figure 1) (5, 35, 36). However, some TLRs, such as TLRs 3 and 4, can signal *via* an additional pathway mediated by TIR-containing adaptor inducing interferon-β (IFN-β) (TRIF). Although this pathway results in the release of NF-κB, it also causes, *via* the IKKε/TANK-binding kinase-1 (TBK1), the phosphorylation of interferon regulatory factor 3 and 7 (IRF3-7), inducing IFN-β expression (35, 36). At the end of these TLR-related molecular cascades, we observe the production and release of several molecular mediators, such as cytokines, chemokines, complement proteins, and enzymes, including IL-1, IL-6, IL-10, IL-11, IL-12, tumor necrosis factor (TNF), TGF, IFN, CCL2, CCL5, CXCL8, and CXCL10, among others (4–6, 15, 35, 36).

Although these mechanisms are the basis of the molecular effects following TLR activation, it is necessary to consider some additional features of the TLR signaling cascade. Taking these findings into consideration, TAK1 activation not only activates the IKK complex but also activates the nemo-like kinase (NLK) (37, 38) as well as the c-Jun N-terminal kinases (JNK) pathway (Figure 1) (35, 36). Similarly, it has been demonstrated that MyD88 can signal through the phosphatidylinositide-3 kinase (PI3K)/Akt pathway, leading to modulation of glycogen synthase kinase 3 β (GSK3β) activity (39, 40). Moreover, it has been suggested that some members of the TLR family, such as TLR2 and TLR4, can also activate the PI3K/Akt pathway *via* a direct interaction with the Ras-related C3 botulinum toxin substrate 1 (Rac1), a member of the Rho family of GTPases (40). However, it is well recognized that the Janus kinase (JAK)/signal transducer and activator of transcription (STAT) (JAK/STAT) pathway respond to a variety of PAMPs/DAMPs and cytokines, including different interleukins and INF-β. However, studies performed by Luu and colleagues (41), and more recently, the work of Song and colleagues (42), have demonstrated that several members of the TLR family can phosphorylate different STAT members, suggesting a direct modulation of the JAK/STAT pathway (Figure 1).

Although we will further discuss the wide signaling pathway involvement, it is important to highlight that an immune response usually triggers the release of inflammatory-related molecules, which can act *via* both autocrine and paracrine mechanisms. Thus, an immune response cannot be considered a linear event, but rather, it should be considered as a cyclic process in which the initial cellular response causes the production and release of secondary molecules that can affect both the same cell and neighboring cells. INF-β is a good example of such a situation. As previously indicated, TLR3 and TLR4 can induce IFN-β production *via* IRF phosphorylation, and this IFN-β can interact with the interferon receptors (INFGR1 and INFGR2), resulting in activation of the JAK/STAT pathway and inducing the expression of further inflammatory-related genes (43, 44). This example exemplifies the complex molecular interactions established during an immune response, and in the following sections, we will highlight the elements that connect the TLR-mediated immune response with the Wnt signaling pathway, a critical molecular cascade in the developing and mature CNS in both physiological and pathological conditions.

### Aβ- and TLR-Mediated Immune/Inflammatory Response

Aβ is a short sequence of 36–43 amino acids that reacts with several biological molecules, including lipids and proteins, which also form Aβ aggregates (23, 45). Although the most common form is Aβ40, Aβ42 is considered to be more reactive and more prone to aggregation (46–49). Aβ has been demonstrated to interact with several members of the TLR subfamily of receptors, including TLR2 and TLR4, inducing an immune response with the subsequent release of pro-inflammatory molecules, including several members of the IL family, such as IL-1β, IL-6, IL-12, TNFα, cyclooxygenase 2, and inducible nitric oxide synthase (4, 46, 48, 50, 51). Importantly, as inflammatory mediators, these molecules can further induce a TLR-mediated immune response, and as previously indicated, because microglia, astrocytes, neurons, and oligodendrocytes express several members of this family of receptors, it is possible to suggest that these cells are not only able to respond to the Aβ challenge but they may also respond to the inflammatory mediators released in response to that initial insult. Indeed, it has been demonstrated that IL-6 levels are significantly elevated in AD, and its increased expression can be achieved *via* both direct production after primary insult on microglia, astrocytes, and/or neurons or as a secondary response to pro-inflammatory mediators such as IL-1β (52–56). Although in a coordinated response there are compensatory mechanisms such as the production of IL-10 and TGFβ that prevent an exacerbated immune response, under chronic pro-inflammatory conditions, similar to conditions of permanent exposure to Aβ in AD, the system will be unbalanced, resulting in an increased production of pro-inflammatory mediators, which worsen the pathological scenario (57). Moreover, it is well established that in the case of microglia, pro-inflammatory signals trigger the activation of this cell, with the subsequent release of additional pro-inflammatory mediators, while the presence of anti-inflammatory molecules (IL-10 and TGFβ) leads to an anti-inflammatory microglial state with the release of neurotrophic factors such as brain-derived neurotrophic factor and glial-derived neurotrophic factor. These factors serve to repair the damage induced initially (57–59). In this regard, it has been demonstrated that activated microglia surround the Aβ aggregates within the brain, suggesting a permanent pro-inflammatory state that contributes to the spread of the pro-inflammatory environment across the brain (60–62).

Similarly, another feature that must be considered is the mitochondrial dysfunction induced by Aβ. This alteration will not only alter the energy balance within the neurons due to impaired ATP production but will also induce an increase in ROS levels, resulting in the production of additional pro-inflammatory mediators, such as IL-6 (63–66). In addition, once released to the extracellular compartment, ROS can further activate the inflammatory pathways in neighboring cells inducing the activation of the NF-κB-dependent pro-inflammatory cascade (67, 68).

Importantly, the inflammatory cascade occurs within a complex cellular molecular network, and under such conditions, it is not difficult to realize that the interaction between the different molecular pathways within the cell is a fundamental feature of the coordinated response against different stimuli. Based on our experience and on the current knowledge regarding neuroinflammation, in the following section, we will describe and discuss the Wnt signaling pathway and its relationship with the TLR-mediated immune/inflammatory response.

## Wnt Signaling Pathway

**Figure 2:**
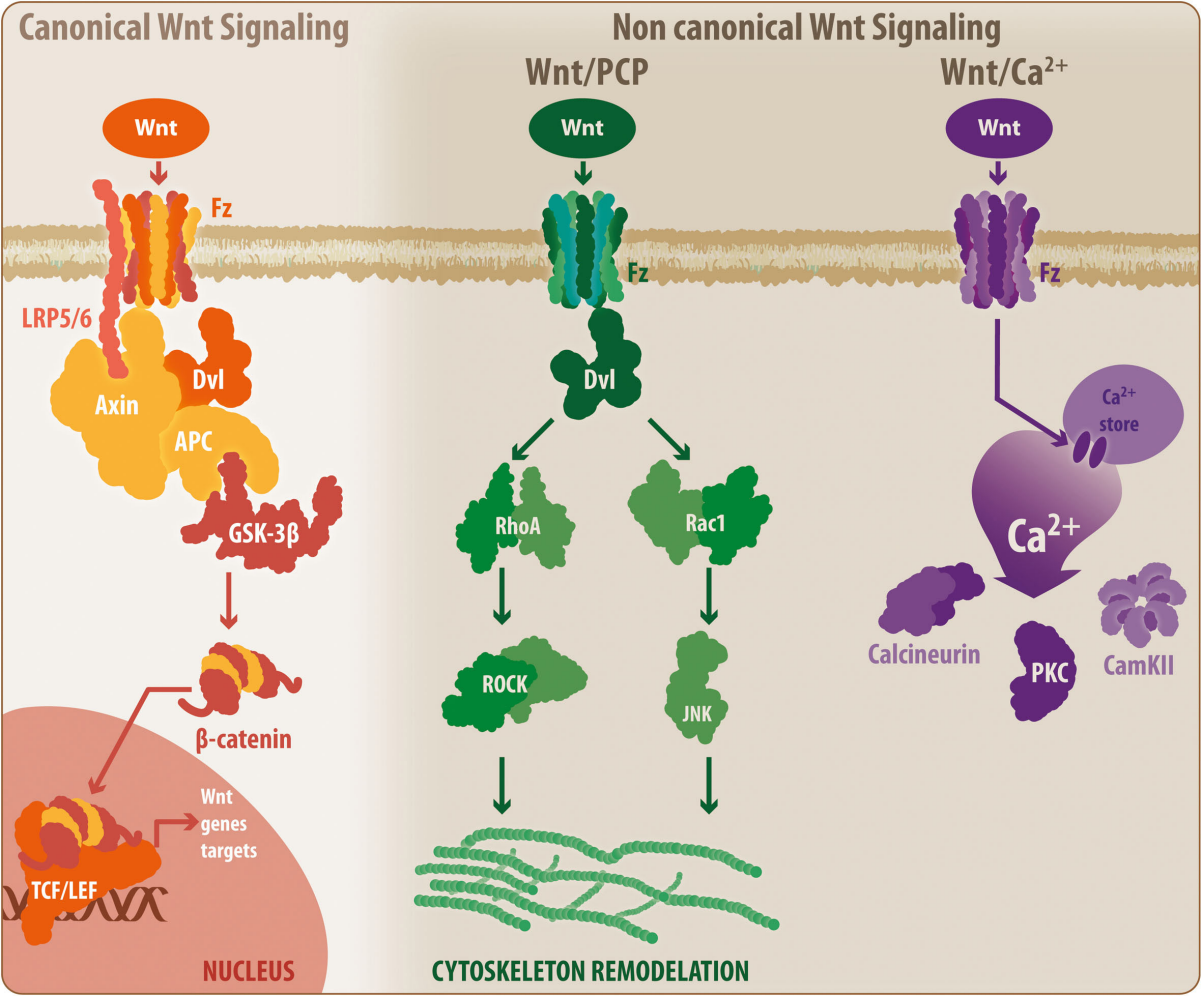
**General overview of the Wnt signaling pathway**. The Wnt signaling pathway can be divided into the canonical Wnt signaling and non-canonical Wnt pathways. During activation, canonical Wnt ligands interact with the Fz-LRP5/6 complex receptor, inducing the activation of Dvl and the recruitment of Axin by LRP5/6. This situation leads to the disassembly of the β-catenin destruction complex, which prevents GSK3β-mediated β-catenin phosphorylation. Thus, β-catenin can translocate to the nucleus where it binds to the TCF/Lef, initiating the transcription of Wnt-related genes. When inactivated, the β-catenin destruction complex remains stabilized, allowing the phosphorylation of β-catenin, inducing its degradation by the proteasome. However, the non-canonical Wnt/PCP pathway requires non-canonical Wnt ligands, which will interact with the Fz receptor and will activate Dvl. However, at this point, Dvl induces the activation of RhoA and Rac, which ultimately will lead to cytoskeletal rearrangement. Similarly, a secondary non-canonical Wnt pathway, known as Wnt/Ca^2+^, can be triggered by non-canonical Wnt ligands. In this case, Fz will induce the release of calcium from intracellular stores, leading to the activation of calcineurin, CamKII, and PKC. Fz, frizzled receptor; LRP, low-density lipoprotein receptor-related protein; Dvl, disheveled; APC, adenomatous polyposis coli; GSK3β, glycogen synthase kinase 3 β; TCF/Lef, T-cell factor/lymphoid enhancer factor; PCP, planar cell polarity; RhoA, Ras homolog gene family member A; ROCK, rho-associated protein kinase; Rac1, Ras-related C3 botulinum toxin substrate 1; JNK, c-Jun N-terminal kinase; PKC, protein kinase C; CamKII, calcium/calmodulin kinase II.

Initially described in *Drosophila*, the Wnt glycoproteins currently constitute a relevant molecular system related to critical physiological events, including cell proliferation and differentiation (69–71). Wnt proteins are highly conserved among different species; in mammals, up to 19 members have been identified (7). Moreover, beyond its physiological roles, several studies, including some conducted by our laboratory, have demonstrated the involvement of this family of proteins in pathological processes of the CNS, such as neurodegenerative disorders (71–76). These Wnt proteins can activate two main pathways: the canonical and non-canonical Wnt pathways (77) (Figure 2).

In the canonical or β-catenin-dependent pathway, Wnt proteins bind to the Frizzled receptor/low-density lipoprotein receptor-related protein 5/6 (Fz/LRP5/6), inducing the activation of the disheveled protein and the interaction between LRP5/6 with Axin. This interaction causes the disassembly of the β-catenin destruction complex consisting of adenomatous polyposis coli, Axin, GSK3β, and casein kinase-1, preventing GSK3β-mediated β-catenin phosphorylation. Next, β-catenin can translocate to the nucleus and bind to the T-cell factor/lymphoid enhancer factor (TCF/Lef) transcription factor to induce Wnt gene expression. In the absence of Wnt proteins, the destruction complex remains active and promotes GSK3β-mediated β-catenin phosphorylation, resulting in proteasomal degradation of β-catenin and blockade of the expression of Wnt target genes (77–79). However, the non-canonical Wnt pathway can be further divided into two additional mechanisms: the Wnt/planar cell polarity (Wnt/PCP) pathway, which requires the binding of Wnt proteins to the Fz receptor to activate disheveled and subsequently Rho and Rac GTPases, inducing JNK activity and leading to actin cytoskeleton modeling (7, 71, 80), and the Wnt/Ca^2+^ pathway in which the binding of Wnt proteins to the Fz receptor causes the release of Ca^2+^ from intracellular compartments, inducing the activation of calcium-related proteins, such as protein kinase C and calcium/calmodulin-dependent protein kinase (Ca^2+^/CamKII) (7, 71, 81).

According to Marchetti and Pluchino (7), Wnt proteins can be categorized according to their ability to induce a specific pathway into two main classes: the Wnt1 subtype, which includes Wnt2, -3, -3a, and -8a; and the Wnt5a subtype, which includes Wnt4, -5a, -5b, -6, -7a, and -11. However, this classification states that the Wnt1 subfamily activates the canonical Wnt pathway, while Wnt5a functions *via* non-canonical signaling, and in some cases, the same Wnt ligand can activate both pathways depending on the physiological/pathological conditions (7). Moreover, it has been established that beyond the external regulation of the Wnt pathways by ligands and inhibitors, the cross talk between both canonical and non-canonical signals can exert a modulatory effect over its counterpart. Furthermore, Wnt pathways also interact with additional signaling pathways, including NF-κB, forkhead box O, Notch, hypoxia-inducible factor 1α, and JNK (28, 82). In this regard, several elements of the Wnt cascade constitute molecular master switches that can be accessed through additional signaling pathways. GSK3β, TCF/Lef transcription factor, and β-catenin are examples of Wnt elements that are able to receive input from additional signaling cascades, which can have a modulatory effect on Wnt pathways. Importantly, these characteristics demonstrate the critical role that Wnt pathways play in several cellular processes, including the pathophysiological mechanisms verified during the development of several CNS disorders, such as neurodegenerative diseases.

## Wnt Signaling and TLR Cross Talk: Toward Neuroinflammatory Modulation

Controlling the immune/inflammatory response in the context of AD has emerged as a fundamental goal to successfully approach treating this disease. However, a general anti-inflammatory drug is not sufficient to resolve the problem, as different interventions are needed to modulate such a response within the CNS. In this regard, the Wnt signaling pathway, which has demonstrated relevant functions in several cell physiological processes, has also important functions as an immune/inflammatory modulator (7, 28, 83).

**Figure 3:**
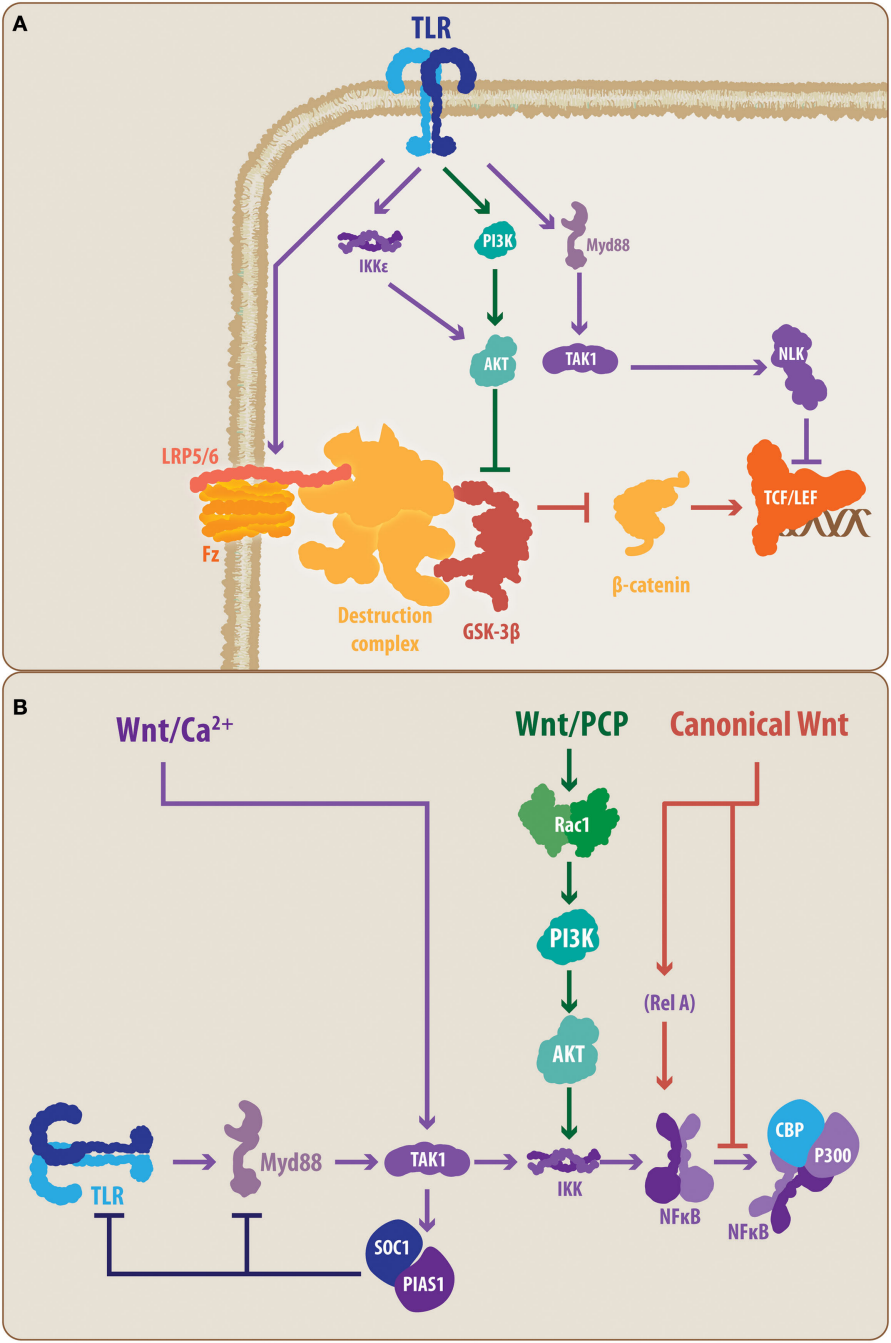
**Proposed molecular talking points between TLRs, Wnt, and nuclear factor-κB (NF-κB)**. The schematic summarizes the suggested interplaying molecular nodes between the different signaling pathways addressed in the present review. **(A)** According to the literature, TLRs can modulate the activity of Wnt signaling at different points. TLRs can prevent the activation of LRP6, supporting the function of the β-catenin destruction complex, thereby promoting the degradation of β-catenin and blocking the expression of Wnt target genes. In contrast, TLRs can lead, through PI3K and IKKε, to the activation of Akt, causing the inhibition of GSK3β. However, TLR signaling can also lead to NLK activation, which can interact with the Lef member of the TCF/Lef transcription factor. **(B)** Similarly, Wnt signaling, through its different pathways, can modulate the activity of the TLRs. The non-canonical Wnt/Ca^2+^ pathway can induce, through TAK1, the activation of SOC1 and PIAS1, causing a reduced expression of TLR signal transducers, such as Myd88. Similarly, the non-canonical Wnt/PCP can interact through Rac1/PI3K/Akt with the IKK complex, modulating the activation of NF-κB. In the case of canonical Wnt signaling, direct modulation of the NF-κB through a Wnt-mediated RelA interaction has been demonstrated. Moreover, it has been suggested that β-catenin can block the interaction between NF-κB and CBP/P300, preventing the transcription of NF-κB-target genes. TLR, toll-like receptor; MyD88, myeloid differentiation factor 88; TAK1, transforming growth factor-β-activated kinase-1; IKK, inhibitory NF-κB kinase; NLK, nemo-like kinase; PI3K, phosphatidylinositide-3 kinase; Akt, protein kinase B; LRP6, low-density lipoprotein receptor-related protein 6; GSK3β, glycogen synthase kinase 3 β; TCF/Lef, T-cell factor/lymphoid enhancer factor; PCP, planar cell polarity; Rac1, Ras-related C3 botulinum toxin substrate 1; SOC1, suppressor of cytokine signaling 1; PIAS1, protein inhibitors of activated STAT 1; CBP, CREB-binding protein, RelA, p65 subunit.

Currently, it is commonly accepted that canonical Wnt signaling acts as an anti-inflammatory mechanism, while the non-canonical pathway has pro-inflammatory activity (7, 28). Several studies have demonstrated the anti-inflammatory activity of the canonical Wnt pathway, and among the mechanisms that explain this effect, the direct interaction with members of the NF-κB transcription factor family, such as RelA, constitutes a relevant mechanism (28, 84, 85). Furthermore, blockade of the canonical Wnt pathway has been demonstrated to promote an inflammatory state, at least in lungs exposed to silica (86). However, activation of the non-canonical Wnt pathway has been demonstrated to promote inflammation and induce the pro-inflammatory state of microglia and to amplify the neuroinflammatory environment caused by Aβ exposure (7, 84, 87). Similarly, through different pathways, including PI3K/Akt, Rac1, and MAPK, non-canonical Wnt ligands induce the activity of the NF-κB transcription factor (88) (Figure 3). However, the non-canonical ligand Wnt5a has also been observed to exert anti-inflammatory activity upon LPS challenge or in bone tissue, and canonical Wnt ligands can induce the pro-inflammatory state in previously activated microglia (89–91). As previously indicated, this situation only confirmed that the physiological and cellular context is important when deciphering the outcome of Wnt pathway activation.

Considering that Wnt pathways can affect the activity of the NF-κB transcription factor, a common end point and a master controller of the TLR-mediated response, the cross talk between the Wnt and TLR pathways appears to be evident (28). However, at this point, some key molecular elements, previously highlighted in the present work, allow the establishment of a closer interplay between the molecular components of both signaling pathways.

Toll-like receptor activation has been demonstrated to downregulate the canonical Wnt signaling pathway. Indeed, TLR4 activation has been shown to prevent LRP6 phosphorylation, which is necessary for Fz-LRP5/6 activity, and block the canonical Wnt signaling at the early steps of the molecular cascade (92) (Figure 3A). However, in macrophages, activation of the non-canonical Wnt pathway, Wnt/Ca^2+^, by the Wnt5a ligand can induce, in a TAK1-dependent manner, the expression of the suppressor of cytokine signaling 1 and of protein inhibitors of activated STAT 1, which causes a decrease in the expression levels of several signal transducers of the TLRs cascade, such as IRAK members and MyD88 (93) (Figure 3B). However, the precise mechanisms of TLR-mediated up-/downregulation of Wnt signaling and vice versa, or if these regulatory mechanisms are confirmed in microglia, astrocytes, and/or neurons, comprise an open field for future research.

Conversely, the MyD88-mediated TLR activity results in TAK1 activation of NLK (Figure 1), which directly interacts with Wnt signaling pathways at the level of the β-catenin-TCF/Lef complex (94) (Figure 3A). Controversially, it has been demonstrated that NLK can positively and negatively regulate canonical Wnt signaling. Indeed, while Ota and colleagues (95) have demonstrated that NLK favors the expression of canonical Wnt target genes *via* Lef phosphorylation, Ishitani and colleagues (96) previously demonstrated a coordinated interaction between TAK1-NLK and the Wnt/Ca^2+^ pathway to antagonize canonical Wnt signaling. Once again, the cell and physiological contexts appear to be determinants for the outcome of such an interaction.

In a similar manner, TLRs, through PI3K, can induce the activation of Akt, a critical kinase able to phosphorylate GSK3β (97). Moreover, it has recently been demonstrated that MyD88-independent TLR signaling activates IKKε/TBK1, which can directly phosphorylate Akt, leading to GSK3β inhibition (98). Importantly, GSK3β constitutes a key protein in the canonical Wnt pathway as its activity induces β-catenin degradation. Consequently, Akt-mediated inhibition of GSK3β can lead to increased activity of the canonical Wnt pathway due to increased levels of stabilized β-catenin, which translocates to the cell nucleus (97) (Figure 3A). In addition, GSK3β activity has also been demonstrated to be relevant for TLR-mediated cytokine production. Indeed, inhibition of GSK3β impairs the ability of NF-κB to bind to the cAMP response element-binding protein (CREB)-binding protein (28, 85, 97). Interestingly, it has also been suggested that this impairment could be mediated by the nuclear β-catenin (Figure 3B). Further confirmation of this interaction mediated through the GSK3β node is provided by studies performed by Li and colleagues (99) on the effects of lithium, a well-known canonical Wnt signaling agonist, which induces the inhibition of GSK3β. In that study, the authors observed that lithium not only reduces the expression of pro-inflammatory mediators, such as IL-6, but it also reduces TLR4 expression in astrocytes. Whether these effects are mediated directly by Wnt signaling or as a part of a secondary mechanism, the convergence around GSK3β further supports the idea that a direct interplay between these pathways is part of the immune/inflammatory response.

## Final Considerations

The immune response constitutes a highly complex mechanism that involves the interaction between several signaling pathways within different cell types. Inflammation is a component of the coordinated immune response, and within the CNS, it has emerged as a critical element because a sustained inflammatory state can further damage the neuronal network. Aβ, an AD molecular hallmark, induces an inflammatory response *via* a TLR-mediated mechanism, and continuous exposure to Aβ perpetuates the pro-inflammatory state. Consistent with these findings, modulation of the inflammatory response is considered a major goal to limit the damage observed in AD. In this regard, the canonical and non-canonical Wnt signaling pathways have demonstrated both pro- and anti-inflammatory activity; however, the precise mechanisms of such a modulatory role are far from clear. During the last decade, several research groups have found relevant evidence suggesting a direct interaction between the Wnt pathways and the TLR-mediated immune response, and although further research is needed to elucidate some controversies observed regarding this interaction, the current knowledge suggests that cross talk between these two molecular cascades can provide insight toward new therapeutic targets in the context of neurodegenerative disorders, such as AD.

## Author Contributions

JZ and NI conceived and wrote the manuscript.

## Conflict of Interest Statement

The authors declare that this research was performed in the absence of any commercial or financial relationships that could be construed as a potential conflict of interest.
